# Adaptation and validation of the Spanish version of the ORTO-15 questionnaire for the diagnosis of orthorexia nervosa

**DOI:** 10.1371/journal.pone.0190722

**Published:** 2018-01-10

**Authors:** María Laura Parra-Fernandez, Teresa Rodríguez-Cano, María Dolores Onieva-Zafra, Maria José Perez-Haro, Víctor Casero-Alonso, Juan Carlos Muñoz Camargo, Blanca Notario-Pacheco

**Affiliations:** 1 Faculty of Nursing Ciudad Real, University of Castilla-La-Mancha, Ciudad Real, Spain; 2 Head of Mental Health, Castilla la Mancha Health Services, Ciudad Real, Spain; 3 School of Industrial Engineers, University of Castilla-La-Mancha, Ciudad Real, Spain; 4 Faculty of Nursing, University of Castilla-La-Mancha, Cuenca, Spain; Kyoto University, JAPAN

## Abstract

The aim of this study was the validation and analysis of the psychometric properties of a Spanish translation of the ORTO-15 questionnaire; an instrument designed to assess orthorexia nervosa behavior. Four hundred and fifty-four Spanish university students (65% women) aged between 18 and 51 years (M = 21.48 ± 0.31) completed the Spanish version of ORTO-15 and the Eating Disorders Inventory-2 (EDI-2). The Principal Component Analysis suggested a three-factor structure for the abbreviated 11-item version of the instrument. The internal consistency of the measurement was adequate (Cronbach's alpha = 0.80). The proposed test demonstrated a good predictive capacity at a threshold value of <25 (efficiency 84%, sensitivity 75% and specificity 84%). Our results support the psychometric properties of the proposed Spanish shortened-version of the ORTO-15 as being a reliable tool for assessing orthorexia nervosa. Its use is expected to greatly contribute to a better understanding of the impact of this disorder in Spain.

## Introduction

Orthorexia nervosa was first defined by Steven Bratman in 1997 [1]. The term Orthorexia, derives from Greek, meaning ὀρθóς (right) and ὄρεξις (appetite) therefore to refer to a “correct or just appetite”[1]. Later, in their book, “Health Food Junkies”, Bratman and Knight described the initial characteristics of people with orthorexia: an obsession for healthy food via adopting a restrictive diet and a focus on food preparation [2].When this obsession for a “pure” diet reaches an extent that could be considered pathological, this is presently referred to as orthorexia nervosa (ON) [3], [4], [5].

According to Bratman, the majority of rigorously-followed diets can lead to ON [2], [6]. Individuals who suffer from this disorder are characterized by their adherence to a restrictive diet; one that strictly avoids certain foods which the individuals consider to be unhealthy, together with the practice of eating rituals (e.g. waiting unusually long periods of time between meals to allow for digestion, based on self-imposed rules regarding food type combinations, or only combining certain foods during certain times during the day) [5], [7]. Different studies have observed that the risk of ON is frequently related to those who opt for a vegetarian or vegan diet [7], [8]. The orthorexic subjects demonstrate characteristics that reflect their specific “feelings” towards food (“dangerous” to describe a product with conservatives, “artificial” for industrially produced products and “healthy” for organic produce). They also demonstrate a strong or uncontrollable desire to eat when feeling nervous, excited, happy or guilty [9]. It is common practice among people with ON to exclusively consume foods originating from organic agriculture which is free from artificial substances, like pesticides and herbicides, thus avoiding transgenic foodstuffs. They are also typically preoccupied with the techniques and materials employed in food manufacturing [10]. Furthermore, this condition may involve psychological and social disorders [5], [11–14]. At the psychological level, these individuals can suffer from feelings of frustration when they transgress or fail to comply with this type of diet [13]. Orthorexic individuals are at risk of social isolation due to their belief that being alone allows them to fully control the entire process of food preparation. Such a belief, for example, may inhibit them from forming relationships with people whose habits and beliefs differ from theirs [5].

Although ON is not, as yet, officially recognized in therapy manuals on mental health disorders, the limited studies that have taken place, to date, with regards this condition indicate that, underlying the obsession for a scrupulously clean diet, is also some type of- psychological disorder [15] which, like many eating disorders, is more strongly related to psychological control than the actual need for any particular food [2], [5], [13]. The pursuit of a healthy diet should clearly not be considered, in itself, as a medical condition. It is when this pursuit leads to a stress-provoking obsession that problems can arise [10]. It has been found that high self-esteem and highly perfectionist tendencies can be characteristics associated with those developing such obsessive behavior [16]. Moroze et al. reported the first diagnostic criteria of the given condition, a four-point criteria based on a clinical case [17]. The first two criteria highlight the obsession for the purity of food, as well as the impact of consuming unhealthy food, feelings of guilt and, in general, the possible social consequences of such obsessive behavior. The last two of the four criteria refer to the diagnosis of the disorder as a medical condition in itself, rather than merely an increase in the severity of some other psychiatric disorder caused by, for example, a food allergy or a religious belief. A year later, Dunn and Bratman published their diagnostic criteria[18], which, in contrast to Moroze et al., comprised two main criteria. The first, concerns the obsession towards healthy foods (dietary restrictions causing, for example, weight loss as well as negative feelings and self-imposed punishments when eating rules are broken). The second criterion is more closely related to the clinical symptoms resulting from the disorder (severe malnutrition, loss of social relations, a distorted impression of one’s own body image and low self-esteem). A person who develops ON may often start out with the desire to improve their health- e.g. treat a given problem or illness, or lose weight, before the diet eventually becomes the “center of their life”[10].

At present there is considerable controversy regarding the classification of ON. Some authors believe it to be an independent disorder [19], others identify the presence of the disorder with obsessive-compulsive traits [9], [20] while the majority of studies attest that the symptoms of ON frequently appear in patients suffering from anorexia nervosa (AN) or bulimia nervosa (BN) [2][14]. Segura–Garcia et al. [21] demonstrated that orthorexic behavior was associated with a clinical improvement in the symptoms of eating disorders. Other studies indicate that the symptoms of orthorexic and anorexic type eating behaviors tend to superimpose, pointing to the possibility of classifying orthorexia as a subtype of anorexia [22].

Two principal assessment tools for the screening of orthorexia symptoms described in the literature are the Bratman Orthorexia Test (BOT) [23] and the test for the diagnosis of orthorexia (ORTO-15) [24]. These were the first tools aimed at identifying orthorexic attitudes. In fact, the latter questionnaire is originally based on the former. Both questionnaires have been translated into several languages and been used in various clinical and non-clinical population groups. However, there is a lack of research on the internal consistency of these instruments. The BOT questionnaire was developed by Bratman [23], based on his own experience both with ON, and as a physician who paid particular attention to the distinction between healthy and unhealthy foods. This questionnaire comprises ten items with dichotomous reply options (yes/no). One of the first studies that used this test to examine the prevalence of ON was carried out by Kinzl [25], who concluded that 12.8% of a sample involving 286 nutritionists presented an ON-tendency (corresponding to four or more affirmative replies). The ORTO-15 questionnaire, proposed by Donini et al. in 2005 [24], combines Bratman’s questionnaire with the Minnesota Multiphasic Personality Inventory (MMPI) [26], one of the most frequently used personality questionnaires in the field of mental health, whose design is focused on the identification of the personality profile and the detection of psychological disorders. Using the ORTO-15 and based on the idea that ON is a disorder characterized by a combination of a phobic behavior towards eating and obsessive-compulsive personality traits, the presence of both these factors indicates a positive diagnosis of ON.

The ORTO-15 test has been adapted and validated in several languages. In 2008, a Turkish version [3] was administered to 994 university students, where confirmatory factorial analysis and a weak internal consistency, with a Cronbach’s alpha of 0.62, resulted in the removal of various items from the original work by Domini, culminating in a final version with a single-factor-structure of 11 items. A shortened version in Hungarian (ORTO-11-Hu) was produced in a study [6] involving a sample of 819 university students and which revealed a positive internal reliability with a Cronbach’s alpha of 0.82, where four items were eliminated. The authors of a study regarding a Polish version [27], presented a nine-item questionnaire with a Cronbach’s alpha of 0.64 in a university sample of 400 students. A recent study featuring a German version [28], of ORTO-15 was administered to a sample of 1029 individuals, among which participants suffering from various conditions (diabetes mellitus type I and II, celiac disease and Crohn’s disease and gastritis) were excluded. The study resulted in a shortened version, comprising nine items in a single-factor structure with a moderate internal consistency of 0.67. To date, this instrument has not been validated for the research of the prevalence of ON in Spain. An adaptation and validation of ORTO-15 in Spanish is therefore timely, as a first step in the study of ON in Spain.

## Methods

### Procedure

A descriptive cross-sectional study was carried out. In total, 800 students (>18) from the University of Castilla-La Mancha were invited to participate in the study. The participants were recruited via informative talks delivered during university lectures at different faculties (Nursing, Law, Chemistry, Computer science and Education). Of these, 315 students refused to participate in the study. Thereafter, 31 participants were excluded due to providing incomplete questionnaires. Therefore, our final sample size was 454 students, aged between 18 and 51 years, who enrolled in the study voluntarily and were requested to complete the online-survey developed using the JotForm platform. The students who refused to provide a response are assumed to be within the same range of conditions as the rest. For ethical reasons, we could not inquire about the causes leading them to reach this decision.The participants received no financial incentive. Written consents were obtained from both the participants and the Ethics Committee of the University Hospital of Castilla-La Mancha, who approved the study according to the ethical guidelines set out in the Declaration of Helsinki, in 2008. The study did not have any exclusion criteria. Our study sample consisted of 295 women and 159 men (64.98% and 35.02% respectively) with mean ages of 21.74 (±4.73) and 20.99 (±3.88) years, respectively. The mean body mass index (BMI), calculated from the weights and heights that the participants reported, was 22.10 (±3.36) for women and 23.67 (±4.06) for men. The aim of the current study is to provide an adaptation-validation of the ORTO-15 in Spanish.

### Instruments

#### Demographic information

Participants’ self-reported sociodemographic characteristics, including age, gender, marital status, educational level and nuclear family, clinical variables such as weight, height, allergies, comorbidities of mental diseases (anxiety disorders, depression and hyperactive disorder), and health habits such as the practice of sport or the consumption of alcohol or cigarettes.

#### Eating Disorder Inventory (EDI-2)

This is a self-reported 91-item questionnaire, answered on a six-point Likert-Type-Scale using a three-point system where ‘sometimes’, ‘rarely’, and ‘never’, are assigned zeros while ‘often’, ‘usually’ and ‘always’ are assigned a score of 1, 2 and 3 respectively. The questionnaire is used to assess eating-disorder symptoms, attitudes and behaviors. It contains 11 subscales: drive for thinness, body satisfaction, bulimia, effectiveness, perfectionism, interpersonal disruption, interceptive awareness, maturity fears, asceticism, impulse regulation and social insecurity. The sub-scale scores can be calculated by simply adding the scores of all the items of each specific sub-scale. The EDI-2 total score ranges from 91 to 546. We used a Spanish version of the scale validated by Corral, Pereña and Seis-dedos in 1998 [29], which showed an internal consistency of 0.83–0.92.

The EDI-2 is widely used in Spain and has proven to be widely accepted as a valid instrument for the accurate diagnosis and detection of the risk of eating disorders (EA) [30–32] among the Spanish population. We chose to use the EDI-2 based on its good psychometric proprieties in both clinical settings and non-clinical samples [33] as well as the possibility it offers of separately assessing different dimensions [34], [35].

#### ORTO-15 questionnaire

The ORTO-15 questionnaire was originally developed in Italian. It is a tool consisting of 15 self-report multiple-choice items with the use of a Likert-Scale (always, often, sometimes, never) to measure three underlying factors related to eating behavior; cognitive-rational (items 1, 5, 6, 11, 12 and 14), clinical (items 3, 7, 8, 9 and 15) and emotional aspects (items 2, 4, 10 and 13). It is used to examine obsessive behavior related to food selection, preparation and consumption habits, as well as attitudes towards healthy food. The lower the score, the higher the indication of behavior or attitudes related to orthorexia is. The Italian group [24] suggested a cut-off score of 40 points, whereby scores below this figure indicate ON related behavior.

For the present study, two experienced and fluent Spanish-as-a-second-language Italian professionals (N.C. and P.G.) translated the ORTO-15 into Spanish. A Spanish professional (F.T.), fluent in Italian as a second language, then translated the Spanish translation back into Italian. Finally, the main investigator of the present study administered the questionnaire to a small group of Spanish students (n = 20) to check the readability and understandability of items and the cognitive equivalence of the translation. The final version of the ORTO-15 was established by consensus and is attached online as Table 1.

**Table 1:** Translation ORTO-15 (version) into Spanish.

1) When eating, do you pay attention to the calories of the food?				
1.-¿Cuándo come, se fija en las calorías de los alimentos?				
2) When you go in a food shop do you feel confused?				
2.-Cuando usted entra a una tienda de alimentos ¿se siente confundido?				
3) In the last 3 months, did the thought of food worry you?				
3.-En los últimos 3 meses ¿pensar en la comida ha sido una preocupación?				
4) Are your eating choices conditioned by your worry about your health status?				
4.- ¿Sus hábitos de alimentación están condicionados por la preocupación por su estado de salud?				
5) Is the taste of food more important than the quality when you evaluate food?				
5.- Para Ud. ¿Es el sabor el principal criterio a la hora de determinar la calidad del alimento?				
6) Are you willing to spend more money to have healthier food?				
6.- ¿Estaría dispuesto a gastar más por una alimentación más sana?				
7) Does the thought about food worry you for more than three hours a day?				
7.- Pensamientos por una alimentación sana, ¿le preocupa más de tres horas al día?				
8) Do you allow yourself any eating transgressions?				
8.- ¿se permite alguna trasgresión alimentaria?				
9) Do you think your mood affects your eating behavior?				
9.- ¿Considera que su estado de humor influye en sus hábitos de alimentación?				
10) Do you think that the conviction to eat only healthy food increases self-esteem?				
10.- ¿Considera que estando convencido de que consume alimentos saludables aumenta su autoestima?				
11) Do you think that eating healthy food changes your life-style (frequency of eating out, friends …)?				
11.- ¿Considera que el consumo de alimentos saludables modifique su estilo de vida (frecuencia restaurante, amigos,. . .)?				
12) Do you think that consuming healthy food may improve your appearance?				
12.- ¿Considera que consumiendo alimentos saludables mejora su aspecto físico?				
13) Do you feel guilty when transgressing?				
13.- ¿Se siente culpable cuando se salta su régimen?				
14) Do you think that on the market there is also unhealthy food?				
14.- ¿Cree usted que en el mercado también hay alimentos poco saludables?				
15) At present, are you alone when having meals?				
15.- En la actualidad, ¿come solo?				
Corrección cuestionario Ortho 15				
ítems				
	**Always** Siempre	**Often** A menudo	**Sometimes** A veces	**Never** Nunca
2,5,8,9	4	3	2	1
3,4,6,7,10,11,12,14,15	1	2	3	4
1,13	2	4	3	1

### Statistical analysis

#### Survey validation

Principal Component Analysis (PCA) was performed to identify the underlying dimensions, measured by the different items in the ORTO questionnaire. The Bartlett’s sphericity test and the Keiser-Meyer-Olkin index have been used to evaluate the positive performance of the PCA. In addition, Horn's Parallel and Velicer’s minimum average partial (MAP) analyses were carried out to extract the optimal number of principal components. The internal consistency of items in the Spanish survey was assessed by means of the Cronbach’s alpha coefficient. Test stability analysis was performed to ensure that the participants correctly understood all the questions formulated in the ORTO-15 survey and that there were no temporal changes in the responses. To this end, the same individuals answered the same survey on two different occasions; the time between test and re-test was 30 days. Cohen’s Kappa coefficient was calculated to measure the level of agreement between the categorical responses.

The predictive capability of the ORTO-15 Spanish version and its threshold value was established through the Receiver Operating Characteristic (ROC) curve and the Youden Index (J) calculation. For this purpose, the same previous sample of individuals completed the Eating Disorder Inventory-2 questionnaire [36]. Those who achieved a higher score in the EDI-2 (cut-point≥110) were assumed to be under the real risk of suffering from an eating disorder [37]. Considering three different possible cut-off points for the Spanish ORTO test, the confusion matrices were constructed. From these, the sensitivity and specificity for each cut-point were calculated. Thereafter, the ROC curve was plotted. The best threshold value maximizes the value of the Youden’s index [38].

To perform all these analysis, R statistical software [39] was used. In addition, the ‘paran’ package [40] was employed to perform the Velicer’s MAP analysis. The confusion matrices were computed through the ‘caret’ package [41]. The significance level for all the cases was established at p<0.05.

## Results

### Item discrimination and internal consistency measurement

When all the survey items are taken into account, the internal consistency of the questionnaire, measured by means of the standardized Cronbach’s alpha, is 0.751. Items 5, 8, 14 and 15 have the lowest item-total correlation values, showing that the correlation between each item and the remaining is weak (see Table 2). When these items are dropped from the original questionnaire, the standardized Cronbach’s alpha for the 11-item questionnaire improves to 0.8, which, according to available research, is an acceptable value [42].

**Table 2:** Reliability analysis of the Spanish ORTO-15 survey.

Variable	Item-Total correlation
**ITEM 1**	0.40
**ITEM 2**	0.31
**ITEM 3**	0.56
**ITEM 4**	0.49
**ITEM 5**	-0.03
**ITEM 6**	0.29
**ITEM 7**	0.44
**ITEM 8**	0.07
**ITEM 9**	0.39
**ITEM 10**	0.54
**ITEM 11**	0.54
**ITEM 12**	0.47
**ITEM 13**	0.53
**ITEM 14**	0.21
**ITEM 15**	0.16

### Principal component analysis

The Bartlett’s sphericity test (p<0.001) and the Keiser-Meyer-Olkin index (KMO = 0.83) showed that PCA is a powerful tool to factorize this dataset.

**Fig 1:**
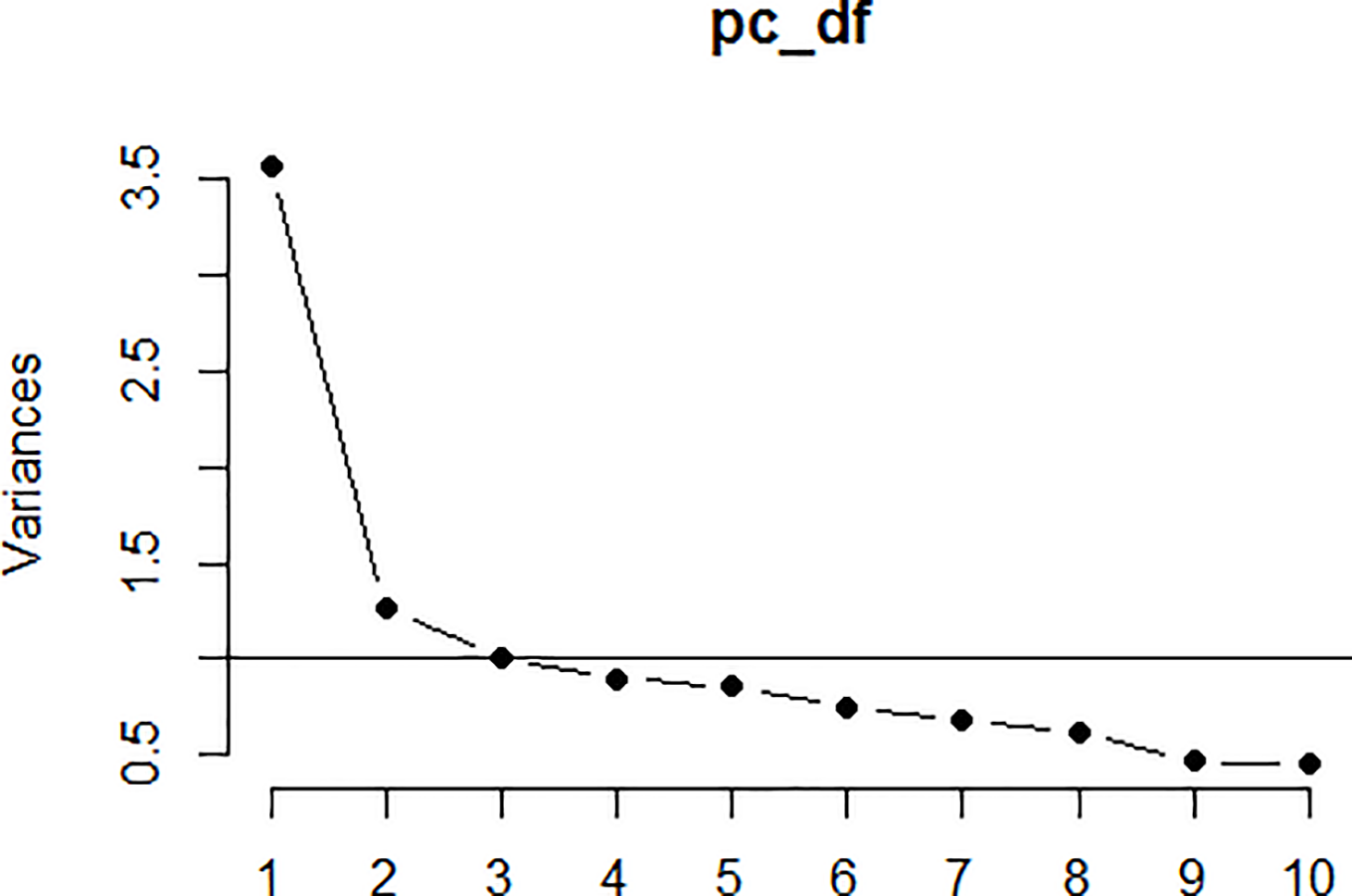
Scree plot.

Fig 1, obtained after performing the principal component analysis, displays a three-factor structure with 11 items, considered to be the most suitable structure of the Spanish ORTO-questionnaire.

This result is based on the Kaiser criterion [43], which recommends choosing those components in which eigenvalues are greater than the unit. In our case, the first three principal components obtained eigenvalues of 3.57, 1.26 and 1.01 and they explain 32.44%, 11.45% and 9.15% of the total variance, respectively. Therefore, the first three principal components explain 53% of the total variance; a very acceptable result.

**Fig 2:**
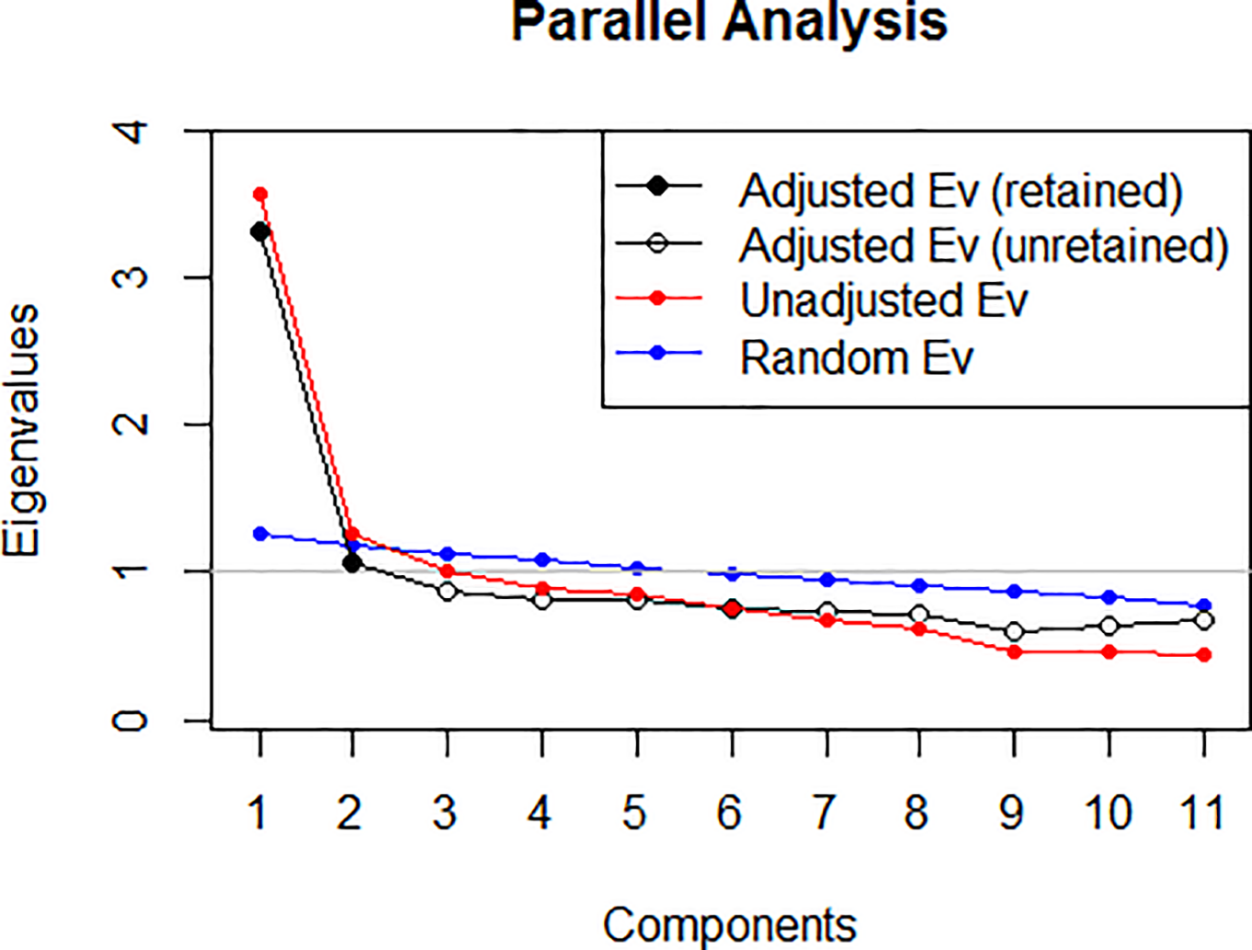
Parallel analysis of the ORTO-11.

However, in order to ensure the best structure, the Horn's Parallel and Velicer’s MAP analysis was performed. A graphical representation of the parallel analysis result is presented in Fig 2 The eigenvalues of the principal components solution suggest two components. The Velicer’s MAP analysis produces an analogous result.

Nevertheless, in the adaptation and validation of the ORTO-questionnaire conducted in other countries, a three-factor structure, in the Turkish and Italian versions [3], [24], and one-factor structure is obtained, in the Hungarian, Polish and German versions [6], [27], [28]. Therefore, we consider the three-factor structure to be more suitable, which is in line with the Italian version from which we have translated the questionnaire.

The loading factors determine the weight of each item in each dimension. The highest scores, after a varimax rotation to simplify the results, have been highlighted in Table 3. Taking into account the composition of each dimension, a label: Rational, Behavioral, and Emotional, was assigned following the Donini’s work [24].

**Table 3:** Loading factors after performing a ‘varimax’ rotation of the original coordinates.

	Rational	Behavioral	Emotional
**ITEM1**	**0.527**	-0.021	-0.063
**ITEM2**	-0.223	**0.609**	0.009
**ITEM3**	-0.135	**-0.562**	0.040
**ITEM4**	**-0.406**	-0.302	0.086
**ITEM6**	**-0.425**	0.1694	-0.148
**ITEM7**	-0.040	**-0.397**	-0.097
**ITEM9**	-0.158	0.098	**0.477**
**ITEM10**	-0.093	0.022	**-0.524**
**ITEM11**	0.065	-0.048	**-0.585**
**ITEM12**	-0.287	0.0892	**-0.326**
**ITEM13**	**0.430**	0.118	-0.0475

### Test stability analysis

The Cohen’s Kappa statistics with a 95% confidence interval were determined for this sample and are displayed in Table 4. Each item was answered on a 1–4 point scale. Moreover, statistical agreement between both answers for all the items is achieved according to the conventional scale established for the Cohen’s Kappa coefficient [27].

**Table 4:** Repeatability analysis for the ORTO-15 survey.

ITEM	Kappa	95% IC
**ITEM 1**	0.97	0.94–1.00
**ITEM 2**	0.98	0.94–1.00
**ITEM 3**	0.95	0.90–1.00
**ITEM 4**	0.97	0.94–1.00
**ITEM 5**	0.91	0.85–0.97
**ITEM 6**	0.95	0.90–1.00
**ITEM 7**	0.97	0.92–1.00
**ITEM 8**	0.96	0.92–1.00
**ITEM 9**	0.92	0.86–0.98
**ITEM 10**	1.00	—
**ITEM 11**	0.97	0.93–1.00
**ITEM 12**	0.95	0.90–1.00
**ITEM13**	0.95	0.91–1.00
**ITEM 14**	0.99	0.96–1.00
**ITEM 15**	0.99	0.96–1.00

### Threshold value determination

Three cut-off points were examined. For each of these (<20, <25, <30) the Accuracy (Ac), Sensitivity (Se), Specificity (Sp), Positive (PPV) and Negative Predictive Values (NPV) and the Youden Index (J) were calculated and are shown in Table 5. J values are between -1 and 1. Furthermore, the closer the index is to the unit, the better the predictive capability of the test is. The best cut-off point was <25.

**Table 5:** Predictive capability of the ORTO-11. Accuracy (Ac), Sensitivity (Se), Specificity (Sp), Positive (PPV) and Negative Predictive Values (NPV) and the Youden Index (J).

Cut Off point	Ac (%)	Se (%)	Sp (%)	PPV (%)	NPV (%)	J
**<20**	97	12	99	14	98	0.11
**<25**	84	75	84	8	99	0.59
**<30**	33	100	32	3	100	0.32

## Discussion

The objective of the present study has been to investigate the psychometric properties of the Spanish version of the ORTO-15 test for the assessment of symptoms of orthorexia nervosa (ON) [24]. This work was motivated by the need for a medical assessment tool, compatible with ON for use on the Spanish population. Our final analysis suggests a 3-factor solution in our Spanish sample, with 11 items (1, 2, 3, 4, 6, 7, 9, 10, 11, 12 and 13) from the original ORTO-15 test included, and four items removed (5, 8, 14 and 15). The internal consistency of this abbreviated, 11-item version (ORTO-11-ES) is found to be acceptable with a Cronbach’s alpha of 0.80.

Since the year 2004, when Donini et al. created ORTO 15, several studies have adapted this questionnaire and have analyzed its psychometric characteristics in various languages. As in our case, most have had to modify the initial questionnaire, eliminating one or more of the original 15 items and modifying the structure of the tool. Based on these studies, the internal reliability of the test shows very variable results, from a Cronbach’s alpha of 0.30 [28] to several studies who place it within more acceptable values, i.e. with a Cronbach’s alpha of 0.82 [6] and 0.81 [44]. Likewise, there are differences in the variability in the number of items eliminated among the different versions, and, in certain cases, there are similarities among the different versions. Some authors point out that these differences in the structure and layout of the test with the removal of some items, may be due to the sociocultural differences between the various countries regarding eating [6]. However, other experts point out that these differences, as such, cannot influence the structure and/or layout of the test, nor the reliability or final sensitivity of the test regarding the detection of the pathology, seeing as, on occasion, the items that are eliminated are more related to the typical symptoms of ON rather than to some of the sociocultural factors that may influence the behaviors associated with the pathology [28].

If we analyze some of the items that have been removed from the questionnaire, in most studies performed, to date, the removal of item number 8 is significant (5, 27, 28). As noted by some authors, the statistical need to remove item 8 should not be justified due to cultural differences and, even less so when considering that all countries removed this item, despite the fact that this information could be deemed very useful. As previous studies have revealed, individuals with ON are highly inflexible regarding their eating habits and do not allow themselves any infringements. The fact that this test is self-reported and not in the context of a clinical interview, may make subjects with orthorexia or with a predisposition to this disorder, not want to face up to or acknowledge their weaknesses or self-discipline regarding their diet or the fact they do not feel that, overall, they are committing a dietary transgression. Also, they do not feel that their diet is strict, but rather that they have merely chosen a healthy way of eating in order to improve their health, in accordance with their own criteria and beliefs. Furthermore, in Spanish, etymologically, the word “transgression” implies an intention beyond breaking rules but, in this case, this is self-imposed and, on the contrary, never imposed by others. It is a choice made according to their own philosophy of life, which could alter the meaning of the item, and therefore the information we were seeking. There is a possibility that the question may not be well worded in the original test, and thus also in the translations thereafter.

Similarly, we find it striking that regarding item 15 (whether the subject eats on his/her own or not), disappears in more sociable cultures, such as those of Turkey and Spain, where the family, the work and the social environment are markedly present during every day mealtimes. This may pose a significant obstacle for people who suffer ON, when attempting to establish the rituals they perform prior to eating which may, obviously, prove difficult in sociable situations. On the other hand, it is undeniable that even in sociable cultures such as our own, the balance between work and family is also changing the eating habits, as well as the timetables and settings of meals [45]. Another aspect worth highlighting is how the different criteria regarding the ON diagnosis could affect the layout of items in the tools for the assessment and/or the detection of ON. In a recent article, Bratman does not declare being either for or against any of the proposed criteria when diagnosing ON, but rather advocates reaching a consensus to include the criteria that derive from the changes in society that mark us with regards lifestyles. In this manner, the aforementioned author proposes to differentiate between what he calls “enthusiasm for eating healthy food”, which has become widespread in current society, and a subsequent intensification of this mind set and/or behavior which can lead to obsessive/compulsive behavior [46]. In this sense, item 1 highlights these differences between criteria and different versions. Thus, the authors of the translation to German and Turkish, with a more representative sample than ours, found no statistically significant justification for keeping this item in their version, whereas our results do include it. Item 1 addresses one of the most debated issues in recent times regarding this disorder in relation to weight watching and counting calories, which, although may be recognized as a source of anxiety in our current society with regard obesity and its impact on health [47], should not be ignored in the detection of ON as being an additional element in the obsessive-compulsive rituals of these individuals.

It is interesting to highlight how our results support those obtained in other validation studies regarding the specific items that have been maintained in all versions (3, 4, 7, 10, 11 and 12). These items reveal those aspects which are strongly related to individuals who are more prone to developing ON, i.e.: excessive time spent on planning meals; excessive worry regarding their health; the fact that they do not mind spending more money to buy food that they consider healthy; the deterioration of social relationships; a positive body image and self-esteem linked to their healthy diet [18].

With regard to the 40 point cut-off proposed by the Italian group in the use of the ORTO-15 questionnaire [24] we propose a cut-off below twenty-five points for the present Spanish ORTO-11. We tested the efficacy, sensitivity, specificity, positive and negative predictive value of three different threshold values for ORTO-11: <20, <25, and <30. Compared to the other two cut-off points, <25 (lower scores refer to more ON features) was considered to be the most appropriate cut-off point for distinguishing between individuals with and without ON. The smaller number of items in our questionnaire may, in part, explain this difference. For other questionnaires with a smaller numbers of items (than those in the Italian study), similar lower cut-offs were proposed; namely, a 27 point cut-off established in a study by a Turkish group [48], and a study using a sample of the Polish population [49], where a score below 24 points indicated a marked preoccupation with the consumption of healthy foods.

Several limitations of this study should be taken into consideration. First, the fact that data were obtained through self-report methods means its accuracy depends on the truthfulness of the respondents and their willingness to share experiences on this sensitive topic. Second, it is necessary to examine the psychometric properties of the ORTO-11-ES in other population sectors in Spain, as this will allow for a better degree of generalization regarding these findings (determined from a sample of Spanish university students). Also, in future studies, the factor structure of the ORTO-11-ES should be confirmed using confirmatory factor analysis (CFA) in an independent sample of Spanish participants. Finally, the present study was set in Spain and despite having an official organization that regulates the Spanish language (Real Academia Española), some words of the translated version should be reviewed when administering the questionnaire in other Spanish-speaking countries. The availability of the Spanish ORTO-11-ES will allow researchers to empirically test its psychometric properties in other Spanish-speaking countries.

## Conclusion

In conclusion, the values obtained in our adaptation and validation of the ORTO-15 questionnaire in Spanish are acceptable. The Spanish ORTO-11-ES could be used to evaluate the scope and comorbidity of ON, as well as to design prevention programs. The questionnaire may facilitate an assessment of the risk of orthorexia within the general population, providing a rapid feedback for health professionals and thus enabling improved control over their own practice. The use of ORTO-11 within a clinical context may facilitate an early and more thorough assessment of a patient’s risk of suffering ON. However, in our opinion, further research is necessary to improve the instrument with new, useful questions for the diagnosis and evaluation of ON.

## Supporting information

S1 File Supporting information. (XLSX)

## References

[1] Bratman S, “Health Food Junkie.,” Bratman S. Heal. Food Junkie. Yoga J 1997;42–50

[2] Bratman S. and Knight D., “Health Food Junkies: Orthorexia Nervosa: Overcoming the Obsession with Healthful Eating,” New York Broadway, 2001.

[3] Arusoğlu G., Kabakçi E., Köksal G. and Merdol T.K. “Orthorexia nervosa and adaptation of ORTO-11 into Turkish.,” Turk Psikiyatri Derg. 2008;19(3):283–91 18791881

[4] Fidan T., Ertekin V., Işikay S. and Kirpinar I. “Prevalence of orthorexia among medical students in Erzurum, Turkey.,” Compr. Psychiatry 2010;51(1);49–54, doi: 10.1016/j.comppsych.2009.03.001 19932826 10.1016/j.comppsych.2009.03.001

[5] Koven N.S., and Abry A.W. “The clinical basis of orthorexia nervosa: emerging perspectives.,” Neuropsychiatr. Dis. Treat 2015;11:385–94 doi: 10.2147/NDT.S61665 25733839 10.2147/NDT.S61665 PMC4340368

[6] Varga M., Thege B.K., Dukay-Szabó S., Túry F., van Furth E.F., Bratman S., et al “When eating healthy is not healthy: orthorexia nervosa and its measurement with the ORTO-15 in Hungary,” BMC Psychiatry 2014;14 (1):59 24581288 10.1186/1471-244X-14-59 PMC3943279

[7] Missbach B., Dunn T.M. and König J.S. “We need new tools to assess Orthorexia Nervosa. A commentary on ‘Prevalence of Orthorexia Nervosa among College Students Based on Bratman’s Test and Associated Tendencies,’” Appetite 2017;108: 521–524 doi: 10.1016/j.appet.2016.07.010 27401768 10.1016/j.appet.2016.07.010

[8] Dell’Osso L., Abelli M., Carpita B., Massimetti G., Pini S., Rivetti L., et al “Orthorexia nervosa in a sample of Italian university population,” Riv. Psichiatr. 2016;51(5) 190–196 doi: 10.1708/2476.25888 27869905 10.1708/2476.25888

[9] Donini L.M., Marsili D., Graziani M.P., Imbriale M., and Cannella C. “Orthorexia nervosa: A preliminary study with a proposal for diagnosis and an attempt to measure the dimension of the phenomenon,” Eat. Weight Disord. 2004; 9 (2):151–157, 200 15330084 10.1007/BF03325060

[10] Catalina Zamora M.L., Bote Bonaechea B., García Sánchez F. and Ríos Rial B. “Orthorexia nervosa. A new eating behavior disorder?.,” Actas Esp. Psiquiatr. 2005;33(1): 66–8 15704033

[11] Park S.W., Kim J.Y., Go G.J., Jeon E.S., Pyo H.J., and Kwon Y.J. “Orthorexia nervosa with hyponatremia, subcutaneous emphysema, pneumomediastinum, pneumothorax, and pancytopenia,” Electrolyte Blood Press. 2011;9 (1):32–37 doi: 10.5049/EBP.2011.9.1.32 21998605 10.5049/EBP.2011.9.1.32 PMC3186895

[12] Bartrina Aranceta J. “Ortorexia o la obsesion por la dieta saludable.,” Arch. Latinoam. Nutr. 2007; 57(4):313–315 18524314

[13] Mathieu, J. “What Is Orthorexia?,” 2005. 10.1016/j.jada.2005.08.021 16183346

[14] Cuzzolaro M. and Donini L.M. “Orthorexia nervosa by proxy?,” Eat. Weight Disord.—Stud. Anorexia, Bulim. Obes. 2016; 21(4): 549–555 10.1007/s40519-016-0310-8 27497695

[15] Muñoz Sánchez R. and Moreno A.M. “Ortorexia y vigorexia:¿Nuevos trastornos de la conducta alimentaria?,” Trastor. la Conduct. Aliment. 2007; 5 (5): 457–482

[16] Barnes M.A. and Caltabiano M.L. “The interrelationship between orthorexia nervosa, perfectionism, body image and attachment style,” Eat. Weight Disord.—Stud. Anorexia, Bulim. Obes. 2017;22 (1):177–184. 10.1007/s40519-016-0280-x 27068175

[17] Moroze R.M., Dunn T.M., Craig Holland J., Yager J., and Weintraub P. “Microthinking About Micronutrients: A Case of Transition From Obsessions About Healthy Eating to Near-Fatal ‘Orthorexia Nervosa’ and Proposed Diagnostic Criteria,” 2015. 10.1016/j.psym.2014.03.003 25016349

[18] Dunn T.M. and Bratman S. “On orthorexia nervosa: A review of the literature and proposed diagnostic criteria,” Eat. Behav. 2016;21: 11–17 doi: 10.1016/j.eatbeh.2015.12.006 26724459 10.1016/j.eatbeh.2015.12.006

[19] Dunn T.M., Gibbs J., Whitney N. and Starosta A. “Prevalence of orthorexia nervosa is less than 1%: data from a US sample.,” Eat. Weight Disord. 2016; 22 (1):185–192. doi: 10.1007/s40519-016-0258-8 26902744 10.1007/s40519-016-0258-8

[20] Brytek-Matera A. “Orthorexia nervosa-an eating disorder, obsessive-compulsive disorder or disturbed eating habit?,” Arch. Psychiatry Psychother 2012;1:55–60

[21] Segura-Garcia C., Ramacciotti C., Rania M., Aloi M., Caroleo M., Bruni A., et al “The prevalence of orthorexia nervosa among eating disorder patients after treatment,” Eat. Weight Disord. 2015;20:161–166 doi: 10.1007/s40519-014-0171-y 25543324 10.1007/s40519-014-0171-y

[22] Barthels F., Meyer F. and Pietrowsky R. “Orthorexic eating behavior1 A new type of disordered eating,” 2015.

[23] Bratman S. Health food junkies: overcoming the obession with healthful eating. 2000.

[24] Donini L.M., Marsili D., Graziani M.P., Imbriale M. and Cannella C. “Orthorexia nervosa: Validation of a diagnosis questionnaire,” Eat. Weight Disord. 2005;10(2):28–32 10.1007/BF03327537 16682853

[25] Kinzl J.F., Hauer K., Traweger C. and Kiefer I. “Orthorexia nervosa in dieticians.,” Psychother. Psychosom. 2006;75(6)395–6 doi: 10.1159/000095447 17053342 10.1159/000095447

[26] Mosticoni R. and Chiari G. “Una descrizione obiettiva della personalità. Il Minnesota Multiphasic Personalità Inventory,” Organ. Spec., 1985.

[27] Brytek-Matera A,. Krupa M., Poggiogalle E. and Donini L.M. “Adaptation of the ORTHO-15 test to Polish women and men.,” Eat. Weight Disord. 2014; 19 (1) 69–76 doi: 10.1007/s40519-014-0100-0 24448996 10.1007/s40519-014-0100-0

[28] Missbach B., Hinterbuchinger B., Dreiseitl V., Zellhofer S., Kurz C. and König J. “When Eating Right, Is Measured Wrong! A Validation and Critical Examination of the ORTO-15 Questionnaire in German.,” PLoS One 2015; 10 (8): e0135772 doi: 10.1371/journal.pone.0135772 26280449 10.1371/journal.pone.0135772 PMC4539204

[29] Corral S., González M., Pereña J., “Adaptación española del Inventario de trastornos de la conducta alimentaria. EDI-2: Inventario de Trastornos de la Conducta Alimentaria.,” 1998.

[30] Castro-Zamudio S. and Castro-Barea J. “Impulsividad y búsqueda de sensaciones: factores asociados a síntomas de anorexia y bulimia nerviosas en estudiantes de secundaria,” Escritos Psicol. / Psychol. Writings 2016; 9(2):22–30.

[31] Rojo-Moreno C., Iranzo-Tatay N., Gimeno-Clemente M.A., Barber-Fons L., Rojo-Bofill M. and Livianos-Aldana L. “Genetic and environmental influences on psychological traits and eating attitudes in a sample of Spanish schoolchildren,” Rev. Psiquiatr. y Salud Ment. 2017 10.1016/j.rpsm.2015.05.003 26163975

[32] Fernández-Delgado A. and Jáuregui-Lobera I.“Variables Psicológicas Y psicopatológicas Asociadas a los trastornos de la conducta alimentaria (TCA) Variables psicológicas y psicopatológicas asociadas con trastornos de la alimentación (ED,” J. Negat. No Posit. Results. RIPED 2016;1(2): 71–80

[33] Garner D.M. “Eating Disorder Inventory 2: Professional manual,” Tea ediciones, 1991.

[34] López C., Educación En D., Gran I., Rey V., Carlos J., Castro-López R., et al “Estudio desciptivo de trastronos de la conducta alimentaria y autoconcepto en usuariosde gimnasios,” vol. 10, no. 2, 2015.

[35] Barthels F., Meyer F., Huber T and Pietrowsky R. “Orthorexic eating behaviour as a coping strategy in patients with anorexia nervosa,” Eat. Weight Disord.—Stud. Anorexia, Bulim. Obes. 2016;1–8. 10.1007/s40519-016-0329-x 27778196

[36] Garner D. Eating Disorder Inventory -2,EDI-2.Proffesional Manual. odessa:Psychological Assessment Resources, 1991.

[37] Correa M.L., Zubarew V.T., Silva P. and Romero M.I. “Prevalencia de riesgo de trastornos alimentarios en adolescentes mujeres escolares de la Región Metropolitana,” Rev. Chil. pediatría 2006; 77(2):153–160

[38] Youden W.J. “Index for rating diagnostic tests.,” Cancer 1950; (3)1: 32–5 10.1002/1097-0142(1950)3:1<32::aid-cncr2820030106>3.0.co;2-3 15405679

[39] Core Team, R: a language and environment for statistical computing | GBIF.ORG vienna, austria, 2016.

[40] Epskamp S. “Path diagrams and visual analysis of various SEM packages’ output.” 2014.

[41] Max Kuhn A., Wing J., Weston S., Williams A., Keefer C., Engelhardt A., et al. “Package ‘caret’ Title Classification and Regression Training Description Misc functions for training and plotting classification and regression models,” 2016.

[42] George D and Mallery P. SPSS, 4th ed. Boston: Allyn and Bacon,.

[43] Kaiser H.F. “The Application of Electronic Computers to Factor Analysis,” Educ. Psychol. Meas. 1960;20(1):141–151

[44] Segura-Garcia C., Papaianni M.C., Caglioti F., Procopio L., Nistic C.G., Bombardiere L. et al “Orthorexia nervosa: A frequent eating disordered behavior in athletes,” Eat. Weight Disord. 2012; 17(4):226–233 10.3275/8272 22361450

[45] Universidad de Barcelona. Facultad de Psicología. and. Grup d’Estudis Alimentaris de la Universitat, Anuario de psicología. 1999; 30(2).

[46] Bratman S. “Orthorexia vs. theories of healthy eating,” Eat. Weight Disord.—Stud. Anorexia, Bulim. Obes. 2017;22 (3):381–385 10.1007/s40519-017-0417-6 28741285

[47] Aranceta-Bartrina J., Pérez-Rodrigo C., Alberdi-Aresti G., Ramos-Carrera N., and Lázaro-Masedo S. “Prevalencia de obesidad general y obesidad abdominal en la población adulta española (25–64 años) 2014–2015: estudio ENPE,” Rev. Española Cardiol. 2016; 69 (6):579–587 10.1016/j.rec.2016.02.009 27133458

[48] Fidan T., Ertekin V., Işikay S. and Kırpınar I. “Prevalence of orthorexia among medical students in Erzurum, Turkey,” Compr. Psychiatry. 2010; 51: 49–54. doi: 10.1016/j.comppsych.2009.03.001 19932826 10.1016/j.comppsych.2009.03.001

[49] Brytek-Matera A., Donini L.M., Krupa M., Poggiogalle E. and Hay P. “Erratum to: Orthorexia nervosa and self-attitudinal aspects of body image in female and male university students.,” J. Eat. Disord. 2016; 4: 1–16 27186373 10.1186/s40337-016-0105-3 PMC4868107

